# Ellenberg's indicator values support prediction of suitable habitat for pre-diapause larvae of endangered butterfly *Euphydryas aurinia*

**DOI:** 10.1371/journal.pone.0179026

**Published:** 2017-06-08

**Authors:** Remigiusz Pielech, Krzysztof Zając, Marcin Kadej, Marek Malicki, Adam Malkiewicz, Dariusz Tarnawski

**Affiliations:** 1 Department of Forest Biodiversity, Institute of Forest Ecology and Silviculture, University of Agriculture, Kraków, Poland; 2 Department of Invertebrate Biology, Evolution and Conservation, Institute of Environmental Biology, University of Wrocław, Wrocław, Poland; 3 Department of Botany, Institute of Environmental Biology, University of Wrocław, Wrocław, Poland; Universita degli Studi di Roma Tor Vergata, ITALY

## Abstract

In spite of the great popularity of Ellenberg’s Indicator Values (EIVs) in plant ecology, animal ecologists seldom use EIVs to address ecological questions. In this study we used EIVs to test their potential usefulness for the prediction of suitable habitat for pre-diapause larvae of the endangered butterfly species *Euphydryas aurinia*. Nine transects crossing grasslands in SW Poland with abundant populations of *E*. *aurinia* were designed. We sampled 76 vegetation plots along the transects. In addition, the presence of the larval webs of *E*. *aurinia* in sampled plots was also recorded. We then calculated the mean community EIVs of light, nitrogen, soil reaction, moisture and temperature for each sample plots. Generalized linear mixed-effects models (GLMMs) were used to assess which factors determine the local occurrence of larval webs of *E*. *aurinia*. We found the larval webs only in 12 plots, while the host plant was present in 39 of the examined plots. The presence of the host plant was the most important predictor in both models including all plots or including only plots with host plants. The other significant predictor was the mean EIV of light, and its importance increased in models considering all plots. We attributed the importance of the EIV of light to the site openness and density of the vegetation layer. A positive relationship between this predictor and the presence of larval webs indicates that sites with looser vegetation, a lower contribution of shrubs and tall herbs and better penetration of photosynthetically active radiation to lower vegetation layers are preferred by *E*. *aurinia* for oviposition. Moreover, the significance of EIV of light may be linked with management practices. Many light-demanding species decline after cessation of mowing as a result of litter accumulation and the dominance of tall herbs. An absence of light-demanding species decreases the community’s mean EIV of light and thus indicates the influence of meadow abandonment.

## Introduction

Since its introduction in the mid-70s, Ellenberg’s Indicator Values (EIVs) have become an extensively used tool in ecological studies. It is especially popular in plant ecology, where EIVs are used to characterize environmental conditions when detailed site-specific measurements are absent. The application of EIVs includes research on vegetation change [1–3], interpretation of ecological gradients [4,5] and describing ecological preferences of both plant species [6,7] and plant communities [8,9]. Some authors, however, have claimed that the interpretation of analyses based on EIVs may be biased due to circular reasoning [10], weak correlations with field measurements [11–13] or inter-correlations between different EIVs [14]. In spite of these reservations, EIVs have enjoyed great popularity and are generally believed to be an important tool in applied plant ecology [15]. On the contrary, animal ecologists seldom use EIVs to address ecological questions, and only a few researchers have tested this approach so far. Significant relationships have been found between EIVs and the diversity of *Sciomyzid* flies [16], butterflies [17–20] and molluscs [21]. In addition, EIVs are sometimes used to characterize the ecological properties of habitats of studied animal assemblages, e.g. ants [22] or molluscs [23]. Some of these studies have highlighted the advantages of the utilization of EIVs in the study of animal autoecology, but this approach is still underexplored and needs more detailed examination.

In this study we used EIVs to test their potential usefulness in the prediction of suitable habitat for pre-diapause larvae of *Euphydryas aurinia*. The species is regionally endangered in many European countries and a great deal of effort has been put so far into supporting its conservation. Some research suggests that host plant abundance is the only important predictor of the presence-absence and abundance of larval webs of *E*. *aurinia* [24]; however, others showed that vegetation structure and habitat management were also important [18,25,26]. We share the latter opinion on the basis of the field observations of *E*. *aurinia* habitats in Poland. The species frequently occurs in meadows with a high density of its host plant; however, there are usually apparently-similar meadows in a close vicinity with abundant populations of known host plants and the absence of *E*. *aurinia*. It is obvious that the ecological niche of the individual species does not overlap exactly with the niche of their host plant [18]. The requirements of either larvae or adults may be limited to a narrower range of ecological conditions than the requirements of the host plants. Understanding this spatial pattern of species distribution at the local scale is essential for its successful conservation. The aim of this work was to examine the relation between the EIVs and the spatial distribution of the pre-diapause larvae of *E*. *aurinia*.

## Material and methods

### Studied species

The marsh fritillary *Euphudryas aurinia* (Rottemburg, 1775) (Lepidoptera: Nymphalidae) is widely distributed in the Palaearctic from Ireland in the West to Yakutia in the East and to North-west China and Mongolia in the South. In many European countries it is reported to be experiencing declines in distribution or population size [27] and the species has become included in Annex II of the Bern Convention on the Conservation of European Wildlife and in Annexes II and IV of the EEC/EU Habitat Directive (92/43 EU of 21 May 1992), and it is legally protected in many European countries. However, both at the level of the Europe and the European Union [28] as well as worldwide [27] it is listed under the least concern category. In Poland, *E*. *aurinia* is listed as endangered in the Polish red list [29].

The species occurs in different types of open or semi-open habitats, such as pastures [30], hay meadows [18,25] or woodland clearings [31]. *E*. *aurinia* has a univoltine life cycle. Adults fly from the third week of May until the third week of June in western Poland. In June (the first three weeks) females lay eggs in batches on the underside of the leaves of the Devil’s-bit scabious, *Succisa pratensis* (probably the only initial host plant for pre-diapause caterpillars in western Poland). Young larvae live together in webs that are built by them directly on the host plant. At the end of the summer or early in the autumn, the caterpillars of the IV instar build stronger webs, located low to the ground, in which they hibernate until spring. They pupate around beginning/mid-May [32,33].

### Study area

All field investigations were carried out within the rural district of Lwówek Śląski in Lower Silesia (SW Poland), covering a total area of approximately 10 km^2^ (50° 57’N, 15° 22’E). The study area is located in the western part of the mountainous region of the Sudetes, at the foot of the massif of the Izerskie Mts. (see [34] for details). The altitude above sea level ranges from 360 to 420 m. The annual average temperature in the research area is 7.5°C, yearly precipitation is ca. 800 mm and the vegetation period is 180 days [35]. The research area is characterized by a large proportion of arable land and forest, which is interposed with patchily distributed fragments of grassland. The different grassland community types within the research area were investigated. Some of the investigated grasslands had been abandoned for up to 10 years, while the others were still managed with various intensities (sporadic to intensive mowing). All grasslands were developed on moderately acid and nutrient poor soils, which had been developed by draining former marshland. According to the phytosociological nomenclature [36,37], the vegetation of the investigated grassland is classified as *Molinion caeruleae* and *Calthion palustris* meadows, while small fragments of *Violion caninae* grasslands may also occur. Some of abandoned meadows are colonized by communities dominated by tall nitrophilous herbs (communities of *Aegopodion podagrariae*) and shrub (communities of *Sambuco-Salicion capreae*) with domination by *Rubus* spp., *Rosa canina*, young individuals of *Betula pendula* and *Populus tremula*. The study area is located in a Special Area of Conservation, Łąki Gór i Pogórza Izerskiego (PLH020102), which was established within the Natura 2000 network. This area is one of the most important refuges of *E*. *aurinia* in south-western Poland [38].

### Data collection and processing

During a few years preceding this study, field surveys had been conducted within the study area. The surveys were aimed at making an inventory of larval webs by *E*. *aurinia*, and they yielded a detailed map of its distribution. We then used this map to design nine linear transects with lengths between 100 and 260 m, which were randomly placed within meadows with a known occurrence of *E*. *aurinia* in previous years. In effect, each transect crossed patches with high densities of *S*. *pratensis*, as a known host plant of pre-diapause larvae in this region, as well as patches without its presence. Along each transect we sampled vegetation plots (2 m × 2 m) placed every 20 meters. In total, 76 plots were sampled in August 2014. Due to the particular phenology of the *Molinion* meadows, we sampled vegetation plots ca. two months after the females of *E*. *aurinia* laid their eggs on the host plants. At the beginning of June, when the eggs are laid, many of the plant species that compose these meadow communities are at an early stage of their development and lack some important diagnostic features. Thus, at this stage it is impossible to differentiate among some species of *Asteraceae*, *Apiaceae*, *Juncaceae* or *Poaceae* families, but the proper determination of all plant species within the sampled plots is crucial to calculate the mean EIVs. Within each plot we recorded all vascular plants and estimated their cover using the Braun-Blanquet scale (r—solitary; +—< 1%; 1—1–5%; 2—6–25%; 3—26–50%; 4—51–75; 5—76–100%). In addition, the presence of the larval webs of *E*. *aurinia* in the sampled plots was also recorded. All collected samples were entered into a Turboveg database [39]. The average means of the EIVs were calculated for each sample [40]. When calculating the community means of the EIVs, we took only the presence/absence data into consideration. We did not used the weights determined from the abundance of each plant species because the abundance of each species could have change between the eggs laid (June) and the vegetation being sampled (August). Finally, the abundance of host plant (*S*. *pratensis*) was transformed into an ordinal scale using the method proposed by van der Maarel [41].

### Statistical analyses

To assess which factors determine the local occurrence of the larval webs of *E*. *aurinia*, generalized linear mixed-effects models (GLMMs) with a binomial error distribution and log-link function were performed. We made two separate models that took into account (1) all the studied plots of vegetation samples and (2) only the plots with the presence of *S*. *pratensis*. For the purpose of both analyses we included six potential predictors of larval web occurrence: abundance of the host plant (variable “Succisa”) and the average community means of the EIVs of “light,” “moisture,” “nutrients,” “soil reaction” and “temperature.” The interpretation of the ecological meaning of the EIVs was presented, e.g. as by Horsák et al. [21]. The transect was used in our analyses as a random effect. Prior to modeling we checked all variables for collinearity using the Spearman rank correlation matrix and the variance inflation factor (VIF). Acceptable levels of correlation were assumed at r_S_ < |0.6| and VIF values below 3 [42,43]. Due to the high correlation between “soil reaction” and “nutrients” and, for the data set based only on relevés with the occurrence of *S*. *pratensis*, between “soil reaction” and “moisture” also (S1 Table), we decided to exclude the “soil reaction” variable from both multivariate models. After this step, we did not find any significant collinearity between the remaining variables (all VIF scores were below 2; see S1 Table). All explanatory variables were standardized to a mean of 0 and standard deviation of 0.5 before inclusion in the models to allow for comparisons of their respective effect sizes [44,45].

To identify factors affecting the pre-diapause larvae of marsh fritillary presence in the study plots we used a model selection procedure based on information theory [46]. We used Akaike Information Criterion (AICc) to select the best reduced model. We ranked all subsets of models according to their ΔAICc values together with the associated weight value (*w*_*i*_). Models with an ΔAICc < 2 were considered to be equally good [46]. To assess whether the final models provided a good fit to the data we calculated the conditional and marginal *R*^2^_GLMM_ [47,48]. The conditional *R*^2^ value showed the proportion of the variance in the raw data explained by the model, including both fixed and random effects, while the marginal *R*^2^ value showed the proportion of the variance explained only by the fixed effects.

The relative importance of each variable was estimated, on a scale 0–1, by summing the AICc weights across all models that included the explanatory variable of interest [46]. To derive the parameter estimates (β) we used model averaging over the 95% confidence set (thus we used all models with sum of Akaike weights ≤ 0.95 [46]). Only beta coefficients in which the 95% confidence intervals (95% CI) did not overlap with zero were considered as significant. Additionally, we used principal component analysis (PCA) for the visualization of vegetation samples in relation to explanatory variables.

All statistical analyses were performed in open source statistical software R (version 3.2.2, http://www.r-project.org/), with the packages: *arm* (version 1.8–6) [49], *lme4* (version 1.1–10) [50] and *MuMIn* (version 1.15.1) [51]. Plots were performed using the packages plotrix (version 3.6) [52] and *ggbiplot* (version 0.55, downloaded from https://github.com/vqv/ggbiplot, December 2015). The variance inflation factor (VIF) was calculated using the ‘vif.mer’ function (downloaded from https://github.com/aufrank/R-hacks/blob/master/mer-utils.R, December 2015) in R.

## Results

The presence of the larval webs of *E*. *aurinia* was found in 12 out of 76 studied vegetation samples (relevés), only on *S*. *pratensis*. The host plant was present in 39 examined plots. The key importance of the presence of *S*. *pratensis* (as the host plant) for the occurrence of larval webs of *E*. *aurinia* was confirmed by the results of the GLMMs. The model selection showed that, taking into account all samples, the two models’ explanations of the presence of larval webs were equally good (Table 1).

**Table 1:** Best generalized linear mixed models ((ΔAICc < 2) describing the presence of the larval webs of *Euphydryas aurinia* in study plots.

No.	Model	df	*R*^2^m	*R*^2^c	AICc	ΔAICc	*w*_*i*_
**All plots**							
1	~ *Succisa* + Light + (1|Transect)	4	0.81	0.81	43.567	0	0.343
2	~ *Succisa* + Light + Temperature + (1|Transect)	5	0.82	0.82	44.840	1.272	0.182
**Plots with host plant presence**							
1	~ *Succisa* + Light + (1|Transect)	4	0.54	0.54	43.369	0	0.299
2	~ Light + (1|Transect)	3	0.36	0.36	45.205	1.837	0.119
3	~ *Succisa* + Light + Temperature + (1|Transect)	5	0.58	0.58	45.271	1.903	0.116

***R***^**2**^**m** –the marginal *R*^2^ value shows the proportion of the variance explained only by the fixed effects, ***R***^**2**^**c** –the conditional *R*^2^ value shows the proportion of the variance in the raw data explained by the model, including both fixed and random effects.

**Fig 1:**
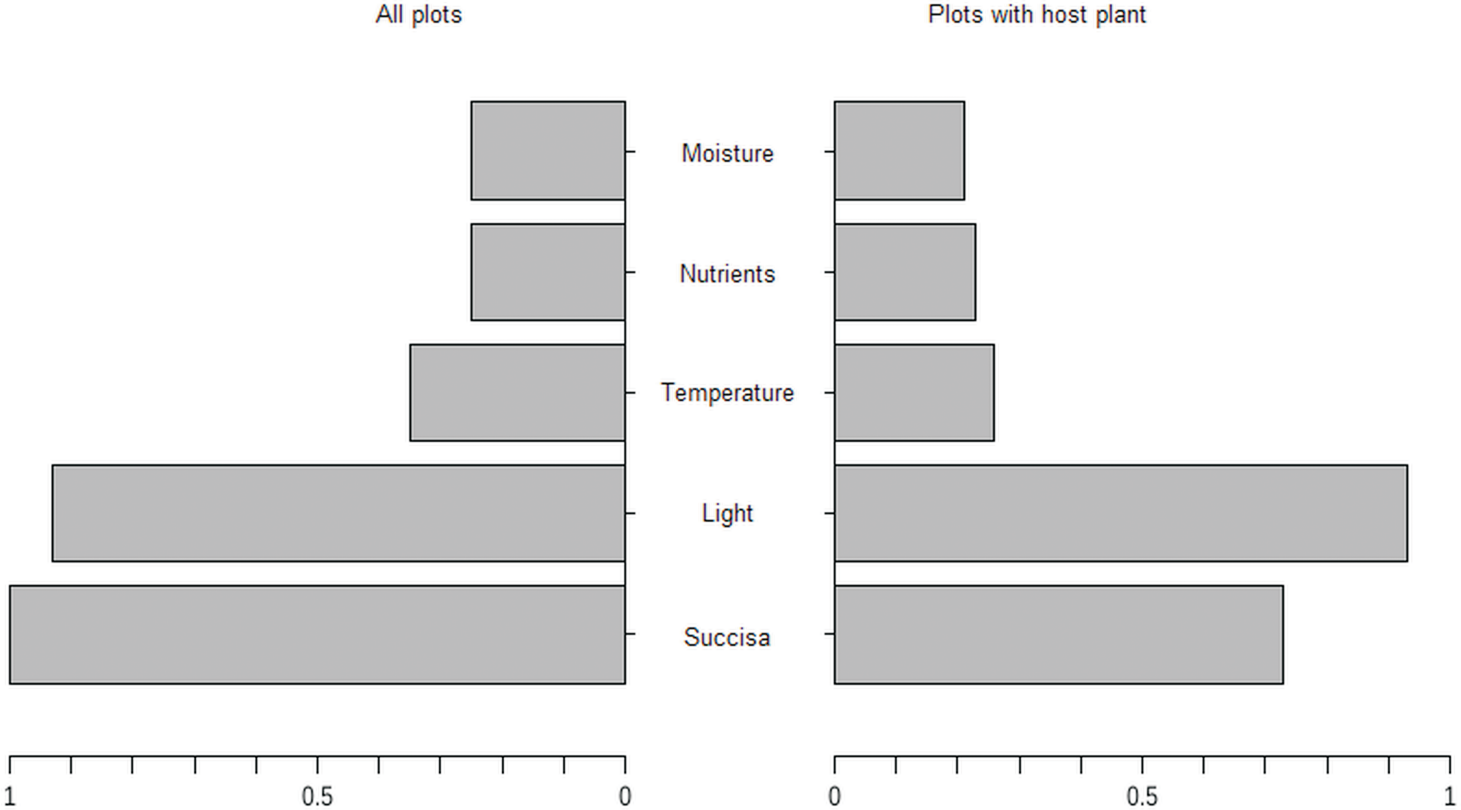
Comparison of the relative variable importance (RVI) used to explain the presence of larval webs of *Euphydryas aurinia* in two data sets: Including all plots and only those with the presence of *Succisa pratensis*. The RVI was computed as the sum of the AICc weights over all models including the explanatory variable.

The best models explained over 80% of the variation and included three explanatory variables. The abundance of the *S*. *pratensis* was the most important variable explaining the presence of larval webs, followed by “light” and “temperature” factors (Fig 1).

**Fig 2:**
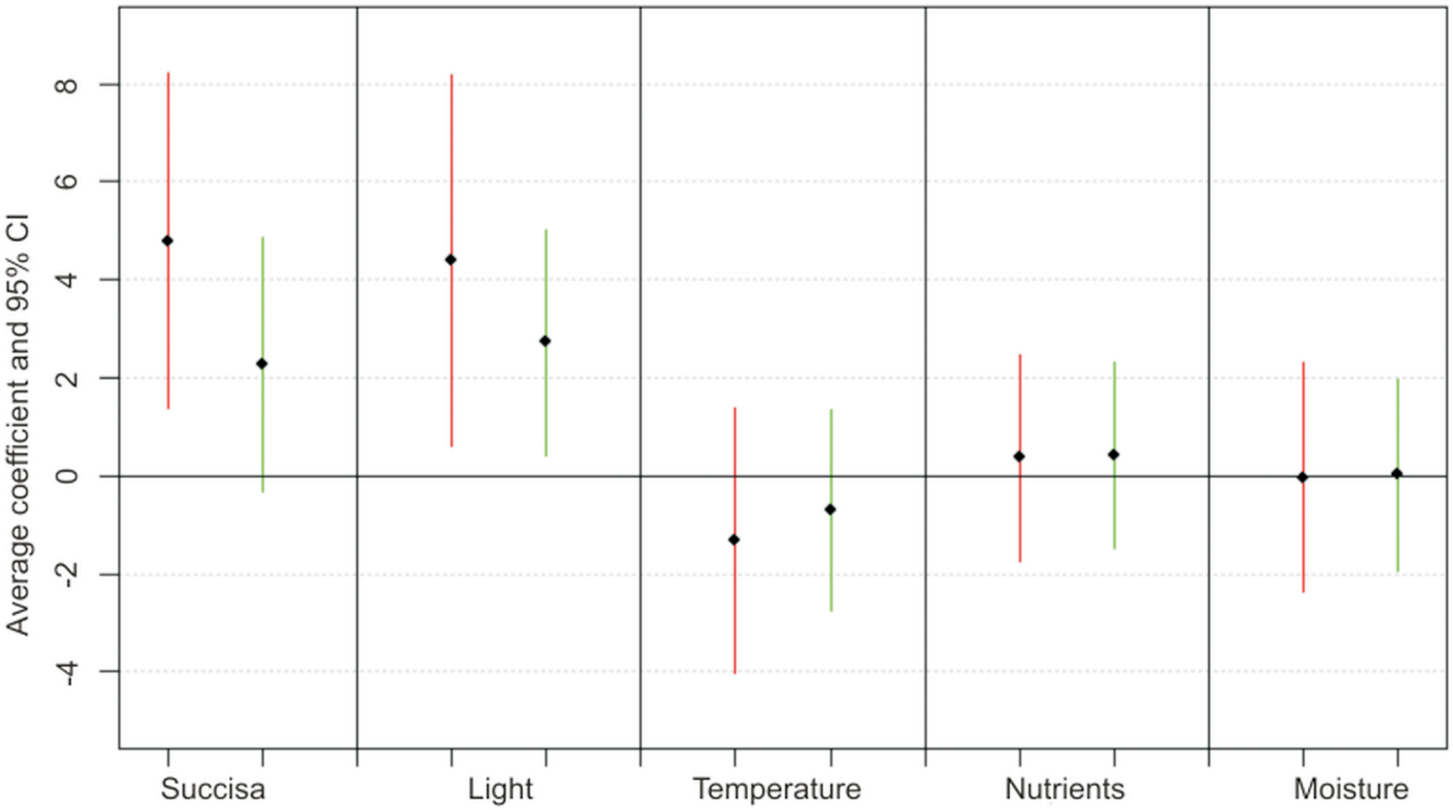
Average parameter estimates and 95% confidence intervals (CIs) for all standardized variables in two data sets. One group includes all plots (red color) and one only those with the presence of *Succisa pratensis* (green color). Parameters were averaged for the 95% confidence set of the models.

Only the positive relation of the first two predictors with the occurrence of larval webs was statistically significant (Fig 2).

**Fig 3:**
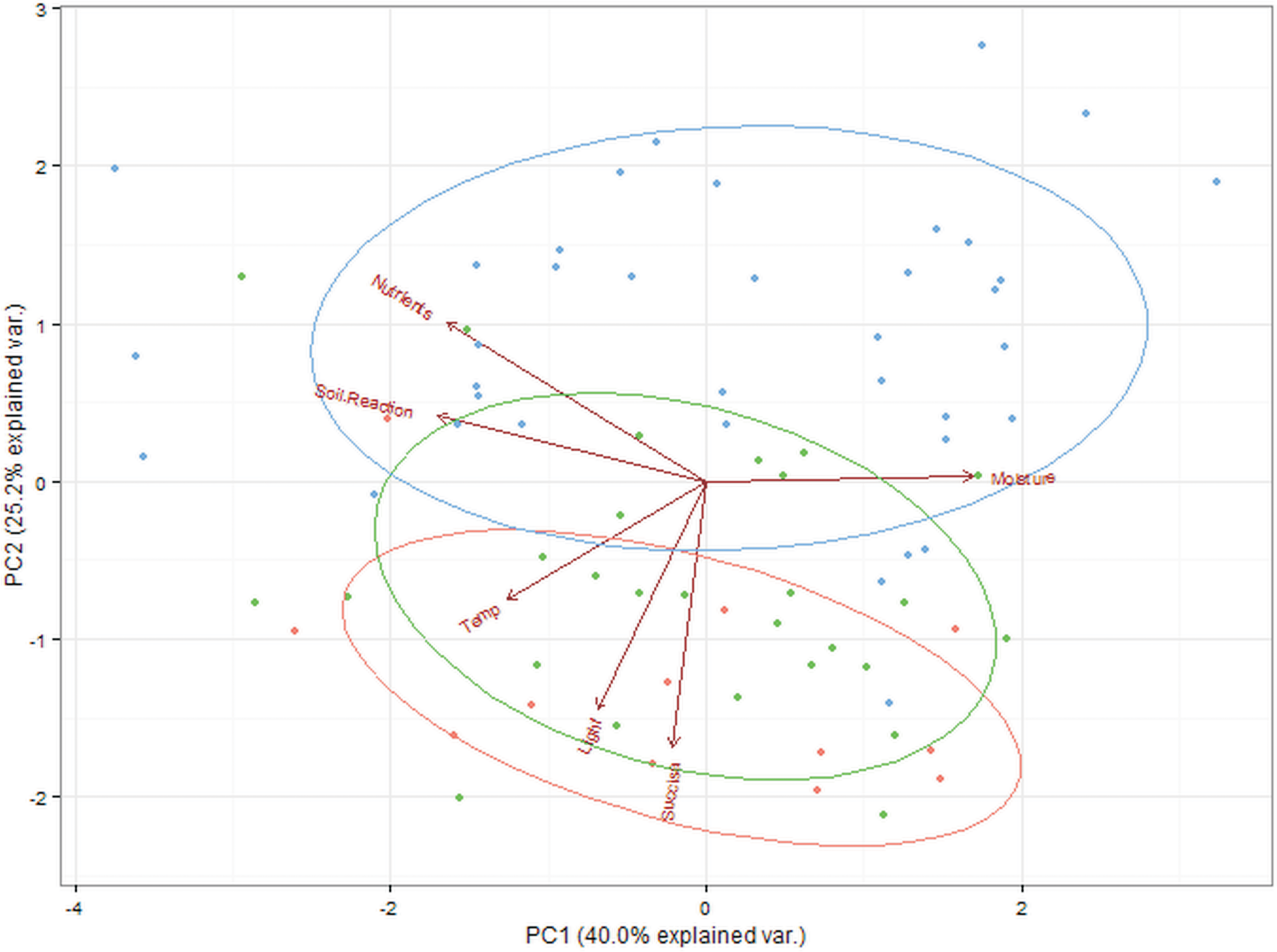
PCA ordination diagram of all 76 study plots of vegetation based on six explanatory variables, with differentiation of the three groups of plots presented with different colors and circled with 95% confidence interval ellipses. Red points and ellipsoid represents plots with *Euphydryas aurinia* occurrence; green points and ellipsoid represents plots with *Succisa pratensis* occurrence and without *E*. *aurinia*; and blue points and ellipsoid represent plots without *S*. *pratensis* and *E*. *aurinia*. Arrows indicate the direction of the explanatory variables. Eigenvalues: PC1–2.397, PC2–1.514.

The same three predictors were present in the best three models explaining the presence of larval webs in the second model for vegetation samples only with the occurrence of *S*. *pratensis* (Table 1). However, in this model the relative importance of the *S*. *pratensis* abundance was much lower, and the most important predictor was “light” (Fig 1), which explained 36% of the variation alone (Table 1). “Light” was also the only variable in the second model with a statistically significant effect, although the larval webs tended to be more frequent with an increase in the abundance of the host plant (Fig 2). For both groups of models, “transect” as a random effect was not a significant term in the GLMM. The distribution of the sampled plots with the occurrence of the larval webs and those without the species, in relation to a gradient of explanatory variables, is shown in Fig 3.

## Discussion

Our research has shown that the distribution of the larval webs of marsh fritillary is closely associated with the presence of *S*. *pretensis*, which seems to be the only initial host plant of the caterpillars in the study population, as in many other regions of central Europe [53]. As we expected, based on the results of other authors [18,26,30,54–56], the abundance of the host plant was an important determinant of the occurrence of pre-diapause larvae. However, our results indicated that after taking into account only samples with the presence of the host plant, the importance of this factor decreased, in favor of the EIV of “light”.

This factor should not be linked with the total solar radiation reaching the site, but rather with the site openness and density of the vegetation layer [21]. A positive relation between this predictor and the presence of larval webs indicates that sites with looser vegetation, decreasing representation of species characteristic of forests and shrubs and better penetration of photosynthetically active radiation to lower vegetation layers are preferred by marsh fritillary for oviposition. Moreover, the significance of the EIV of “light” may be attributed to management practices. Many light-demanding species decline after the cessation of mowing as a result of litter accumulation and the dominance of tall herbs [57–61]. The absence of light-demanding species decreases the community mean EIV of “light” and thus indicates the influence of meadow abandonment.

Based on this ecological interpretation, our results are consistent with those reported by other researchers, who showed that the females of *E*. *aurinia* chose individual *S*. *pratensis* in open vegetation structures that were fully exposed to the sun and surrounded by lower vegetation as host [18,25,26]. Larvae of *E*. *aurinia* may increase their growth rate behaviorally by sun basking, thus microclimate conditions shaped by the looser structure of the vegetation and better access for the light may be particularly important in the case of a low temperature environment [62]. Moreover, the rosette of *Succisa* leaves is situated close to the ground and is easily accessible for egg-depositing females in an open vegetation structure [25]. The accessibility and sun-exposure of the host plants are, next to visibility, the most important factors determining the female oviposition in the Mediterranean subspecies *E*. *a*. *provincialis* [63].

The presence or abundance of the host plant is of course the most important factors determining habitat quality for *E*. *aurinia*. However, there are also some other environmental requirements that have to be met to enable its successful reproduction. Assessing habitat quality only on the basis of the number of host plants may be somewhat misleading. For example, individuals of *S*. *pratensis* in abandoned populations have higher growth rates and produce more flower heads per plant (in spite of higher mortality rates and lower seedling establishment) [64]. As we stated above, these abandoned sites are at the same time unfavourable for *E*. *aurinia*. Thus, various variables related to the vegetation structure and dynamics can both fine-tune the prediction of the insect’s distribution and habitat quality assessment. As we show in this research, EIVs may be a useful tool in that field.

## Supporting information

S1 Table Spearman correlation coefficients between explanatory variables and variance inflation factors (VIFs) for variables in final models. Significant correlations (p<0.05) are indicated in bold.
(DOC)
