# Supplementary material for: Ellenberg's indicator values support prediction of suitable habitat for pre-diapause larvae of endangered butterfly Euphydryas aurinia
Source: PLoS One. 2017 Jun 8;12(6):e0179026. doi: 10.1371/journal.pone.0179026 (PMC5464623; doi:10.1371/journal.pone.0179026)
Supplement: S1 Table — Significant correlations (p<0.05) are indicated in bold. (DOC) [file pone.0179026.s001.doc]

| Variables | Spearman rank correlation | | | | | | VIF |
| --- | --- | --- | --- | --- | --- | --- | --- |
|  | Light | Temperature | Moisture | Soil Reaction | Nutrients | Host plant |  |
| **All plots** | | | | | | | |
| Light | - | **0.305** | 0.018 | 0.186 | 0.077 | **0.356** | 1.113 |
| Temperature | **0.305** | - | **-0.327** | **0.271** | **0.232** | 0.195 | 1.639 |
| Moisture | 0.018 | **-0.327** | - | **-0.414** | **-0.463** | -0.082 | 1.909 |
| Soil Reaction | 0.186 | **0.271** | **-0.414** | - | **0.687** | 0.066 | - |
| Nutrients | 0.077 | **0.232** | **-0.463** | **0.687** | - | -0.157 | 1.384 |
| Succisa | **0.356** | 0.195 | -0.082 | 0.066 | -0.157 | - | 1.232 |
| **Plots with *Succisa pratensis* only** | | | | | | | |
| Light | - | -0.085 | 0.145 | -0.112 | 0.014 | 0.234 | 1.039 |
| Temperature | -0.085 | - | **-0.403** | 0.244 | 0.166 | 0.203 | 1.722 |
| Moisture | 0.145 | **-0.403** | - | **-0.601** | **-0.545** | -0.023 | 1.905 |
| Soil Reaction | -0.112 | 0.244 | **-0.601** | - | **0.685** | -0.053 | - |
| Nutrients | 0.014 | 0.166 | **-0.545** | **0.685** | - | -0.076 | 1.382 |
| Succisa | 0.234 | 0.203 | -0.023 | -0.053 | -0.076 | - | 1.261 |
